# A novel computer modeling and simulation technique for bronchi motion tracking in human lungs under respiration

**DOI:** 10.1007/s13246-023-01336-2

**Published:** 2023-10-03

**Authors:** Byeong-Jun Kim, Hyo Yeong Ahn, Chanhee Song, Dongman Ryu, Tae Sik Goh, Jung Sub Lee, Chiseung Lee

**Affiliations:** 1 Department of Biomedical Engineering, Graduate School, and University Research Park, Pusan National University, Busan, 49241 Republic of Korea; 2 Department of Thoracic and Cardiovascular Surgery, School of Medicine, Biomedical Research Institute, Pusan National University, Pusan National University Hospital, Busan, 49241 Republic of Korea; 3 Medical Research Institute, Pusan National University, Busan, 49241 Republic of Korea; 4 Department of Orthopaedic Surgery, School of Medicine, Biomedical Research Institute, Pusan National University, Pusan National University Hospital, Busan, 49241 Republic of Korea; 5 Department of Biomedical Engineering, School of Medicine, Pusan National University, Busan, Republic of Korea; 6 Biomedical Research Institute, Pusan National University Hospital, Busan, 49241 Republic of Korea

**Keywords:** Computer modeling and simulation, In silico medicine, Lung bronchi (or tumors) motion tracking, Patient-specific finite element thorax model, Ogden’s hyperelastic model

## Abstract

In this work, we proposed a novel computer modeling and simulation technique for motion tracking of lung bronchi (or tumors) under respiration using 9 cases of computed tomography (CT)-based patient-specific finite element (FE) models and Ogden’s hyperelastic model. In the fabrication of patient-specific FE models for the respiratory system, various organs such as the mediastinum, diaphragm, and thorax that could affect the lung motions during breathing were considered. To describe the nonlinear material behavior of lung parenchyma, the comparative simulation for biaxial tension-compression of lung parenchyma was carried out using several hyperelastic models in ABAQUS, and then, Ogden’s model was adopted as an optimal model. Based on the aforementioned FE models and Ogden’s material model, the 9 cases of respiration simulation were carried out from exhalation to inhalation, and the motion of lung bronchi (or tumors) was tracked. In addition, the changes in lung volume, lung cross-sectional area on the axial plane during breathing were calculated. Finally, the simulation results were quantitatively compared to the inhalation/exhalation CT images of 9 subjects to validate the proposed technique. Through the simulation, it was confirmed that the average relative errors of simulation to clinical data regarding to the displacement of 258 landmarks in the lung bronchi branches of total subjects were 1.10%~2.67%. In addition, the average relative errors of those with respect to the lung cross-sectional area changes and the volume changes in the superior-inferior direction were 0.20%~5.00% and 1.29 ~ 9.23%, respectively. Hence, it was considered that the simulation results were coincided well with the clinical data. The novelty of the present study is as follows: (1) The framework from fabrication of the human respiratory system to validation of the bronchi motion tracking is provided step by step. (2) The comparative simulation study for nonlinear material behavior of lung parenchyma was carried out to describe the realistic lung motion. (3) Various organs surrounding the lung parenchyma and restricting its motion were considered in respiration simulation. (4) The simulation results such as landmark displacement, lung cross-sectional area/volume changes were quantitatively compared to the clinical data of 9 subjects.

## Introduction

The number of patients with lung diseases has been increasing rapidly in recent years due to the aging population, COVID-19, viruses, and air pollution. In addition, the development of medical devices has led to the widespread adoption of minimally invasive surgery (MIS) using endoscopes such as bronchoscopy and thoracoscopy. One of the most important aspects of minimally invasive surgery is to accurately identify where the lung lesion moves during patient breathing (during bronchoscopy) and lung contraction (during thoracoscopy) [1, 2].

Various studies have been proposed to accurately identify the location of lung lesions that change frequently. Radioactive chemical injection and water-soluble chemical injection are the representative examples. These are very useful methods, but there are some fatal disadvantages. That is, in the case of the former, excessive radiation exposure to patients and medical staff is caused by the need to use CT frequently during surgery [3, 4]. In addition, in the case of the latter, there is a high possibility of missing the location of the lung lesion during surgery due to the high diffusion speed of the injected material [5–9].

Alternatively, studies have been conducted on tracking the location of lung lesions using computer modeling and simulation [10–17].

The studies used CT or MRI data to model the lungs using various programs, such as Insight Toolkit, MeshMixer, Mimics, and 3D Slicer. Then, the parameters of the lung material were selected using elastic and hyperelastic materials for each organ. The finite element mesh was created using tetrahedral and hexahedral elements with HyperMesh, IA-FEMESH, Ansys Mesh, and TetGen software.

Both contact conditions between the lung parenchyma and surrounding organs were used, and boundary conditions were applied to various locations of the trachea, lung, and rib. Loads were defined by applying PV curves or negative pressure during lung inhalation and exhalation [18]. The analysis conditions were linear, nonlinear finite element models, inverse finite element models, and inverse nonlinear finite element models. Finally, the verification method was to create landmarks on the target organs using 3D-CT and FEM, 4D-CT and FEM, and to qualitatively and quantitatively clinically verify the deformation distance, position, movement, vector error, and volume error.

However, their papers mainly analyzed the behavior of the lung parenchyma. The contact conditions with the surrounding organs (diaphragm, ribs, mediastinum, and pulmonary vessels) were not considered. The material properties of the lungs were assumed to be elastic and hyperelastic. And the results and clinical verification were not described in detail step by step, from the modeling of the human respiratory system to the verification of bronchial movement tracking.

Therefore, in this paper, we propose a new computer modeling and simulation technique for tracking the movement of the lung bronchi (or tumor) during breathing using patient-specific finite element (FE) models based on CT scans and hyperelastic models. This technique is designed for successful minimally invasive surgery (MIS). We also validate the accuracy of FEM by comparing it with clinical data.

The originality of this study is summarized as follows: (1) early diagnosis of lung diseases, (2) accuracy of lung tissue behavior, (3) consideration of more factors, (4) details on each step and validation. This is a new approach that has not been seen in other similar studies.

First, we developed a biomechanical model of the human lung by predicting the changes in respiratory motion of the bronchi (or tumor). This model can be used to early diagnose lung diseases. It can be quickly diagnosed and implemented at the patient’s bedside. In other words, it will be used as a diagnostic tool [19].

Second, we performed respiratory simulation of the human body lung based on a hyperelastic constitutive model and compared the results with clinical data. That is, in order to simulate the material behavior of the human lung, which exhibits hyperelastic material properties, we used various hyperelastic constitutive models, such as the Mooney-Rivlin, Ogden, Van der Waals, neo-Hooke, Yeoh, polynomial, and reduced polynomial models. Based on this, we determined the material parameters by adopting a constitutive model that best simulates the biaxial tension-compression diagram of the body lung reported in the literature [20].

**Fig. 1:**
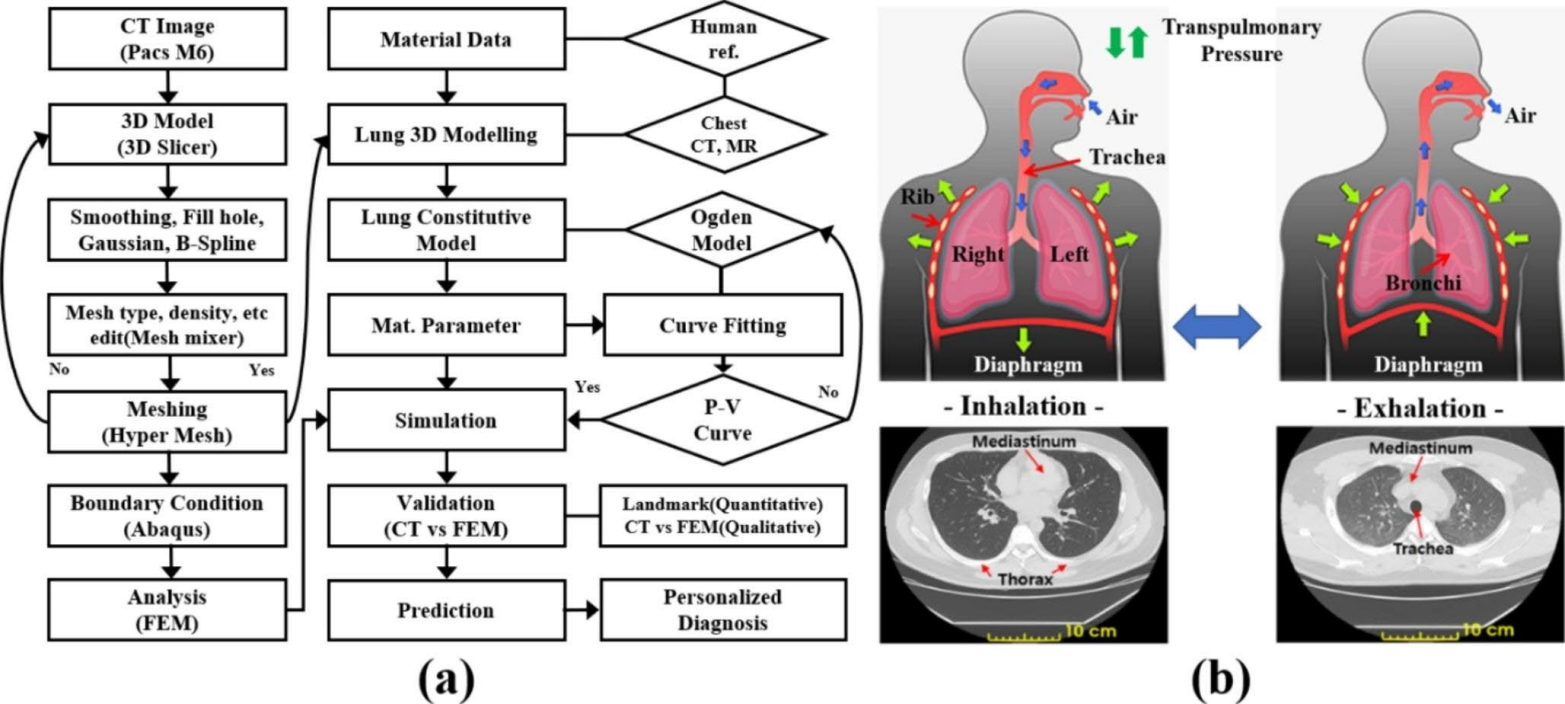
(**a**) Computational framework patient-specific modeling procedure of three-dimensional (3D) geometries and finite element validation method for human respiratory system; (**b**) Schematic of the inhalation and exhalation procedure in the human respiratory system

**Fig. 2:**
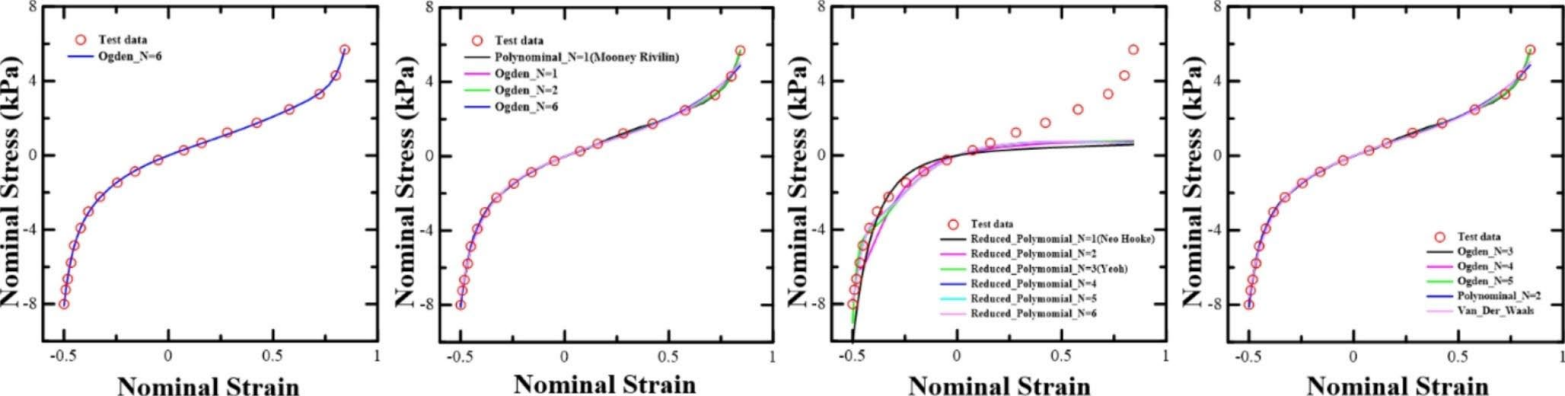
15 cases hyperelastic material models were evaluated for strain energy, including simulated experimental data

Hyperelastic material models were used based on the experimental test data made by Zeng et al. Fifteen models, including experimental data, were evaluated as the strain energy (Fig. 2). In this paper, we used the Ogden_N6 model, which is most satisfactory for stability and the least squares method.

Third, a model that considers all contributing factors, including the patient’s specific material properties, shape, load, and boundary conditions, is needed for accurate tracking of lung deformation [21]. In this study, a model that included all of the following organs, which had not been considered previously, was considered. Among the factors involved in pulmonary respiration, this study considered the rib, mediastinum, and diaphragm and the heart, various tissues, and blood vessels and applied transpulmonary pressure that had the greatest effect, that is, the differential pressure between the bronchi and alveoli. Considering these factors, results of lung deformation were presented as quantitative values for each location through the comparison of computed tomography (CT) and FEM results.

Fourth, using CT data, a 3D model was extracted during inhalation and exhalation. The model was created using the surface interpolation technique, which enabled numerical analysis within the actual and minimal error ranges. Physical property values were defined based on the results of existing tensile experiments on the human lung and bronchi [20]. Fifteen types of isotropic hyperelastic models of the lung parenchyma and elastic properties of the bronchi were used. The data model that most consistently matched the stability and curve-fitting results was selected. Each element was created as a four-node linear tetrahedron C3D4. To ensure the accuracy of the boundary conditions and loads, transpulmonary pressure, which is the largest measure for specific patient classification and an important index of pulmonary respiration, was defined as a time load condition according to respiratory pressure. The analysis method was the inverse nonlinear FEM method. This method was used after overlapping the CT data and simulation results of real human lungs [22]. The accuracy of the lung deformation simulation was verified by comparing the area, volume, and specific person’s lesion bronchi trajectory with landmark points and plane slices for quantitative and qualitative errors during the inhalation and exhalation phases of the lungs [Fig. 1(a)]. Therefore, we further verified the usefulness of this technique for predicting the location of lung lesions through a mathematical model-based finite element analysis that predicts the physical behavior of lung respiration. Finally, through clinical verification, we attempted to perform a rapid and accurate minimally invasive surgery using lesion-location tracking.

## Methods

In general, soft tissues under loading have a nonlinear stress-strain relationship related to the application of finite elasticity. Previous studies by Carter et al. and Werner et al. have shown that the physical material elastic properties of lung tissue can be mainly modeled using the linear Hooke’s Law, which describes the linear relationship between force (stress) and deformation (strain). However, Pinart, Freed et al. recently reported that the Mooney–Rivlin model (Mooney 1940) is appropriate for describing the viscoelasticity of the lungs [12]. In their study of 14 lung cancer patients, they found that almost all of the deformations were concentrated in the lower and middle lobes of the lungs, and quickly dissipated within a short distance from the border surface of the diaphragm and lungs. A similar deformation pattern was also observed in a study of 152 lung cancer patients [18].

In this study, simulations were conducted from inhalation to contraction during exhalation. A large deformation occurred during contraction from inhalation to exhalation. The sex, image dimensions, number of voxels, and surface area of the CT image of nine selected patients at the end of exhalation are shown in Table 1. There were two women and seven men. The image dimensions were 512 mm × 512 mm. The 3D segments of the CT image before simulation and FEM after simulation were compared and quantified. The CT and FEM data showed similar values proportionally, without significant differences. Hyperelastic material models were used based on the experimental test data made by Zeng et al., with a Poisson’s ratio (ν) of 0.43 [21]. Data curve fitting was performed using finite element software package (Abaqus 2020, Dassault Systems, USA) to evaluate the hyperelastic material behavior with strain energy. Fifteen models were evaluated for strain energy. These models included experimental data. (Fig. 2). There were 11 unstable models. Four stable models matched the curve-fitting results with experimental values: Polynominal_N = 1 (Mooney–Rivlin), Ogden_N = 1, Ogden_N = 2, and Ogden_N6. Additionally, when the nominal strain value of each graph was 0.5 or higher, the most consistent models for curve fitting with the test data in the curve where the nonlinearity rapidly occurs were Ogden_N = 3, Ogden_N = 4, Ogden_N = 5, and Ogden_N = 6.

As a result of the calculation by the least squares method, the Ogden_N = 6 model had the smallest least squares value. Therefore, in this paper, we used the Ogden_N6 model, which is most satisfactory for stability and the least squares method. Additionally, the elastic modulus and Poisson’s ratio were used as elastic materials for the properties of the bronchus on the basis of previous studies (Table 2) [21].

**Table 1:** Nine Selected patient models from Segment Statistics at the End of Exhalation [23]

End of Exhalation (EE)		Image dimension	Number of voxels [voxels]		Surface area [mm^2^]	
Subject no.	Gender	512 × 512 pixels, 0.7657 × 0.7657 *mm* (In-plane pixel)	CT	FEM	CT	FEM
Case1	M		3,276,836	3,656,222	154,015	171,507
Case2	F		1,373,182	1,502,589	107,703	116,509
Case3	M		2,787,532	3,115,214	215,985	176,705
Case4	M		1,467,890	2,018,560	162,462	153,770
Case5	M		1,755,663	1,624,783	180,655	144,746
Case6	M		3,628,030	3,121,571	286,269	214,621
Case7	F		2,454,117	2,483,207	186,080	139,919
Case8	M		1,272,570	1,398,171	166,262	148,465
Case9	M		154,521	1,425,773	161,453	125,472

### Anatomy and physiology of lung respiration

Breathing is divided into inhalation and exhalation. Inhalation is called active movement because it is based on muscle contraction (diaphragm) involved in the inhalation movement, and exhalation is called passive movement because it occurs during the relaxation of the external intercostal contraction during exhalation.

The diaphragm, which has the greatest influence on breathing, has the main role of repeating contraction and relaxation to control the size of the thoracic cavity and change its internal pressure to enable breathing. During inhalation, it contracts and moves down, increasing the volume of the thoracic cavity and reducing internal pressure. This inflates the lungs where atmospheric pressure acts. On the other hand, during exhalation, it relaxes and moves upward. As a result, the volume of the thoracic cavity decreases and the internal pressure increases, which is higher than atmospheric pressure. This pressure is called transpulmonary pressure, and it forces air out of the lungs [Fig. 1 (b)] [24].

**Table 2:** Material Properties of respiratory system [25–29]

Hyper-elastic	Lungs		
Ogden N = 6	M1: 2.801	A1: 1.827	D1: 0.289
	M1: -3.480	A1: 2.393	D1: 0
	M1: 1.135	A1: 2.918	D1: 0
	M1: 1.396	A1: -5.631	D1: 0
	M1: -1.304	A1: -6.082	D1: 0
	M1: 0.167	A1: -7.007	D1: 0
Elasticity	Trachea	Mediastinum	Rib
E	5 kPa	5.87× 10^−3^	5000
ν	0.44	0.4	0.4

### Implementation of lung biomechanical modeling

**Fig. 3:**
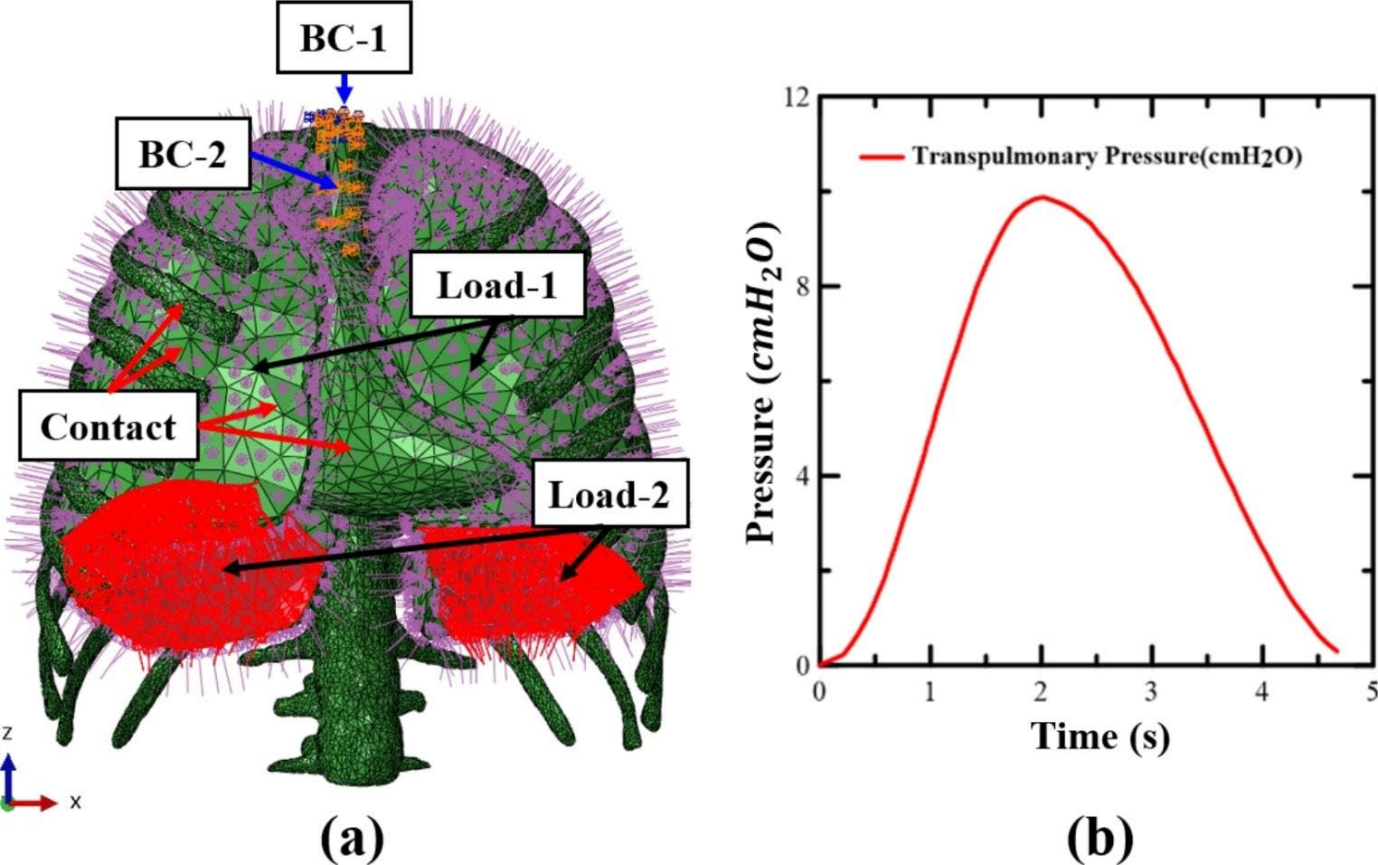
(**a**) Load, boundary, and contact conditions of the respiratory system, including the rib, lung, mediastinum, and diaphragm; (**b**) Transpulmonary pressure measurement curve using spirometry experiment; Exhalation (0–2 s), Inhalation (2–5 s)

To obtain accurate results for finite element analysis, a 3D model of the lungs, bronchi, mediastinum, and ribs in the chest is required. A 3D model was extracted with 420 slices using a CT image processing program (3D Slicer Image Computing Software, USA) from nine experimenters before and after respiration using a medical image storage system (Picture Archiving and Communication System, Siemens). Images were collected and exported in accordance with the Digital Imaging and Communication in Medicine (DICOM) protocol. The parameter configuration for the CT scan was as follows: 18 s scan, 100 kV tube voltage and automatic tube current of 128 mA. The generated Stereolithographic file (STL) was loaded into the Autodesk Meshmixer to recover the non-dimension geometry [30, 31]. The extracted 3D model had a rough surface and was sporadically abnormal due to low image resolution. To solve this, we filled the holes and smoothed the surface. For the finite element analysis, the 3D models must be converted into finite element meshes (FEMs). Because mesh elements have a significant effect on the accuracy, convergence, and speed of calculations, a more appropriate number of elements and non-uniform rational B-spline (NURBS) models are required. A 3D geometrically simplified shape with an anatomical form is also needed to prevent an overlapping area of elements [32]. Figure 3(a) shows a representative FE mesh element model among the experimenters. A 3D model was created using a program for mesh element work (Meshmixer 2017, Autodesk, USA) and (HyperMesh 2019, Altair, USA). Considering the many curvatures of the lungs and bronchi, the type and number of elements most suitable for the shape were applied. As a result of referring to many biomechanical simulation studies, tetrahedral elements were determined to be more appropriate than hexahedral elements [Fig. 1(a)] [33, 34].

### Loading and boundary conditions

Among the factors involved in pulmonary respiration, this study considered the rib, mediastinum, diaphragm, heart, various tissues, and blood vessels, and applied transpulmonary pressure, which had the greatest effect, that is, the differential pressure between the bronchi and alveoli. The goal was to verify the amount of deformation applied to the lung by applying a load to the external surface and understand the movement of the lung bronchi (or tumors).

In Fig. 3(a), using Abaqus 2020 (Dassault Systemes, USA), a finite element commercial program, the X, Y, and Z axes were restricted from rotation and translation in the BC-1, which is the upper part of the trachea. In the BC-2 region, contraction and relaxation, similar to small cymbals, were observed on CT imaging. The Z-axis rotation and translational motion were constrained to be possible, and the X and Y axes were constrained to prevent rotation and translational motion.

Contact conditions were imposed on the mediastinum and lungs, and between the rib cage and lungs, with frictionless contact conditions, so that the movement of the rib cage and mediastinum during respiration can be known. As in the surface of the lungs in Load-1, when the load on the lungs is fully inflated, a maximum of 10 (0.98 [kPa]) pressure is applied to the entire outer surface except the diaphragm for a total of 5 s under the assumption of normal breathing as in Fig. 3(a) and (b), exhalation for 0–2 s, and inhalation for 2–5 s. In Load-2, the load on the part with the diaphragm was applied by acquiring the displacement of the diaphragm in the exhaled state on the CT scan of each experimenter.

The full Newton static analysis, which is a numerical analysis method, was used to obtain the Abaqus/Standard nonlinear equilibrium equation solution, and because the lung has a large deformation, a nonlinear large deformation model was used. Elements of the trachea were smaller than those of the lung parenchyma, therefore, they were designated as master and slave surfaces, respectively. The simulation took 43 min of analysis time on a workstation with 64 cores and 128 threads.

## Results

**Fig. 4:**
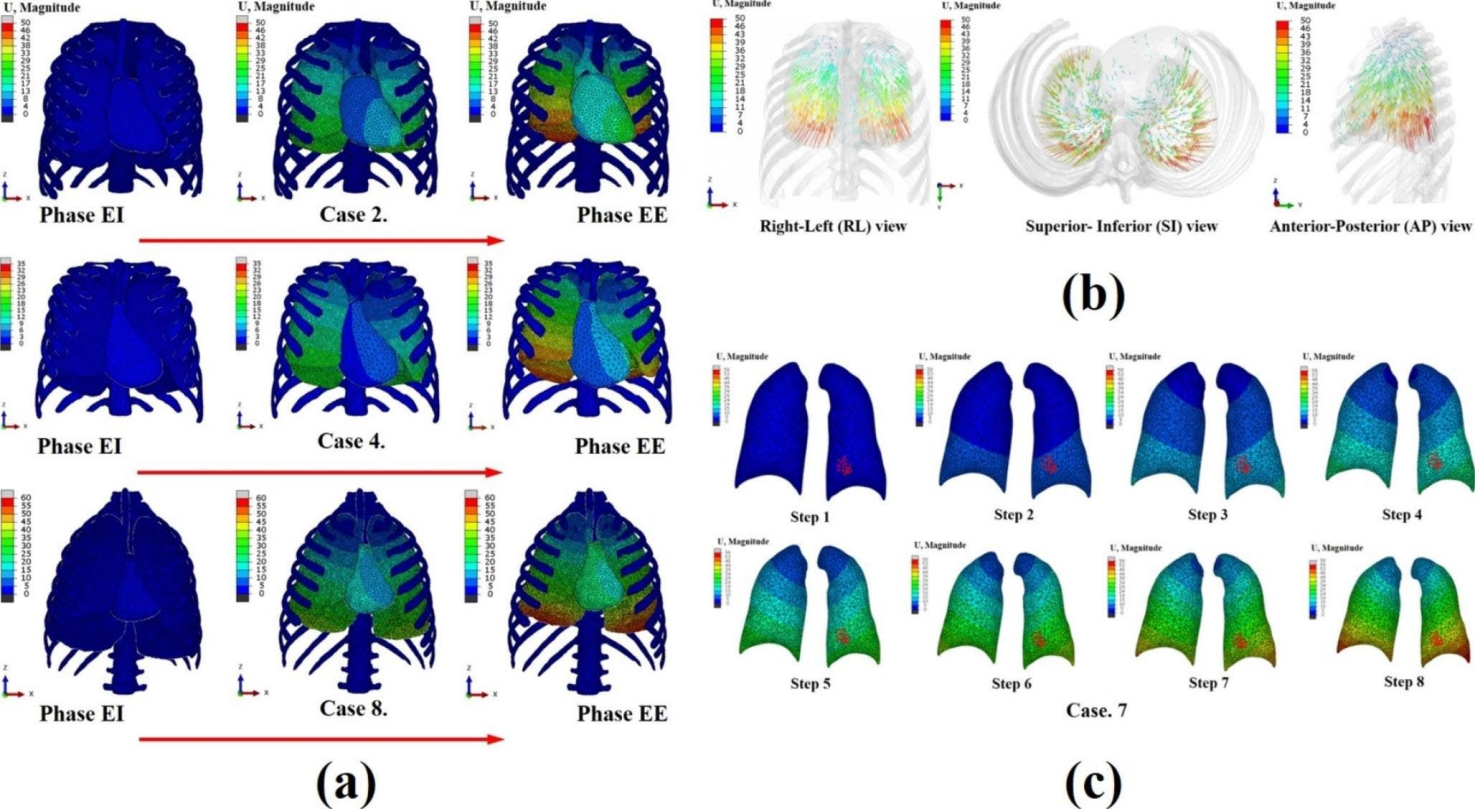
(**a**) Result of specific human biomechanical finite element method simulations; lung and mediastinum deformations during the whole respiratory breathing for Cases 2, 4, and 8; (**b**) Lung FEM simulation vector field result of Case 3; (**c**) Qualitative analysis of human specific biomechanical simulation; lungs bronchi (tumor) landmark deformations during the 0s ~ 2s of breathing for human case. 7

The clinical data CT model and simulation analysis results of the FEM were quantitatively and qualitatively verified. After constructing an FEM as [Fig. 4(a)] using the end-exhalation (EE) and the end-inhalation (EI) lung CT of nine experimenters, the volume of the lung and mediastinum was deformed according to the transpulmonary pressure, the ribs supporting the lung by the diaphragm, and the surface pressure. The lungs were also deformed by contact with the mediastinum. In addition, the exhalation state was implemented, and the change in displacement resulting from time and lesion tracking was evaluated. Furthermore, to verify the proposed method, the simulation results of the series were quantitatively and qualitatively compared with the exhaled state and lung CT images. Several landmarks were placed on the lung lesion bronchi to indicate the movement of the lesion during lung respiration [35].

### Finite element analysis results (displacement and vector field)

The simulation results obtained by inputting the material properties, load, and boundary conditions were analyzed using the finite element discretization equation and expressed as a displacement vector (U). The displacement field and amount of deformation could be viewed quantitatively. Figure 4(b) shows the human lung in Case. 3. The displacement of the vector field result can be seen, with the arrows indicating the final positions and directions of all nodes of the finite elements after the simulation was completed. The length of the arrows is proportional to the displacement vector coefficient. When exhalation was complete (complete contraction), the upper lobe of the lung hardly moved, and deformation occurred mainly at the bottom of the diaphragm. As a result of the FEM simulation analysis by creating landmarks (RL: right lower, LL: left lower) in the lungs of nine humans, the number of landmarks and nodes (triangles), computation time, and superior-inferior direction mean displacement of the diaphragm are shown in Table 3. The maximum displacement of the diaphragm can be confirmed on the right-posterior (RP) and left-posterior (LP) sides. It is also possible able to notice RP side movements that are slightly larger than LP side movements, consistent with physiological anatomy.

**Table 3:** Lung Lobes of the specific patient, the number of landmarks, element number and simulation time result

Subject no		Number of landmarks	Element	Computation time
Case1	RL	29	246,783	20 min
Case2	RL	28	1,045,295	60 min
Case3	RL	31	868,267	51 min
Case4	LL	30	403,689	28 min
Case5	LL	28	596,615	31 min
Case6	RL	33	499,492	30 min
Case7	LL	29	511,069	30 min
Case8	LL	28	741,115	42 min
Case9	LL	22	615,616	38 min

### Landmarks evaluation at end of inhalation (EI) and the end of exhalation (EE)

**Fig. 5:**
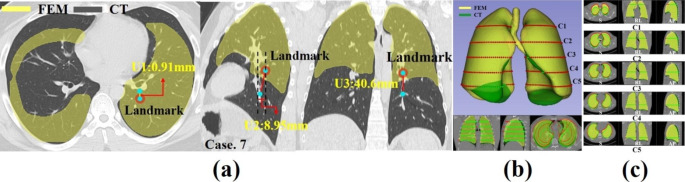
(**a**) Comparison and measurement of biomechanical finite element simulation results in the exhalation state and CT in the inhalation state; (**b**) CT and FEM 3D Model Overlap; (**c**) L/R, A/p, S/I direction slice view

The proposed FEM biomechanical simulation model was validated in eight steps between the EI and EE states for the location of bronchi (or tumor) and lung deformation by the mediastinum and diaphragm. Among the numerical analysis conditions of the nine human models, the tumor location of Cases 1, 2, 3, and 6 was right lower (RL) and that of Cases 4, 5, 7, 8, and 9 was left lower (LL). The number of landmarks was 22 ~ 33 points; elements, 4,036,890 ~ 1,045,295; and the calculation time of each model was at least 20 ~ 60 min (Table 3). As shown in Fig. 4(c), a region in which tumors are likely to occur is indicated in Case 7’s model. The results of the expiratory finite element method (FEM) analysis were subjected to CT and compared with the coordinates of the lung bronchi. As a result, as shown in Table 4, the three-direction (left/right [L/R], superior/inferior [S/I], and anterior/posterior [A/P]) displacements and errors of the CT and FEM simulations of all case models were calculated. In particular, Fig. 5(a) show the displacement in the L/R, S/I, and A/P directions of how much the FEM-simulated tumor in the EE state moved from the existing tumor coordinates on the CT in the EI state of Case 7. Therefore, as a result of evaluating the nine cases in a similar manner, the displacement in the S/I direction was the largest, followed by A/P and L/R. The error was the lowest in the S/I direction, followed by the L/R and A/P directions. In conclusion, the CT model and FEM simulation model were consistent between 1.1 and 2.67%.

**Table 4:** Average landmark lung error analysis result

Subject no.		CT mean Displacement (mm)			Simulation Mean displacement			Error (%)		
		L/R	A/P	S/I	L/R	A/P	S/I	L/R	A/P	S/I
Case 1	RL	3.38	8.59	38.70	3.40	8.62	38.00	0.5	0.3	1.8
Case 2	RL	-14.80	0.48	27.39	-14.50	0.50	28.01	2.0	4.1	2.2
Case3	RL	-5.93	15.10	27.90	-5.91	15.55	27.6	0.3	2.9	1.0
Case4	LL	0.42	9.50	35.89	0.40	9.35	34.95	4.7	1.7	2.6
Case5	LL	13.74	-1.27	23.7	13.57	1.22	23.6	1.2	3.9	0.4
Case6	RL	8.84	11.65	31.59	8.74	12.01	31.52	1.1	3.0	0.2
Case7	LL	6.35	16.25	39.82	6.42	16.55	39.99	1.1	1.8	0.4
Case8	LL	-5.89	3.52	35.23	-5.59	3.42	34.97	5.0	2.8	0.7
Case9	LL	-4.66	10.36	78.59	-4.52	9.98	78.05	3.0	3.6	0.6

### Overlap of 3D model results of CT and FEM

The results of the FEM 3D model overlapped with the CT 3D model and quantitative verification method are as follows: Fig. 5(b) shows the cross-sectional view (L/R, S/I, and A/P) of the 3D model results of the CT and FEM simulations of the contractions to the end of exhalation from Case 1 of 9 cases. The 3D models were overlapped and offset by 28.6 mm in a total of five layers to the upper and lower parts of the lung.

Figure 5(c) shows the superior-inferior cross-sectional area of C1 through C5. The left ellipse of each figure is the right human lung, the right is the left lung of the ellipse, and the small circle between them is the bronchus. The measured results showed the sex of each case, volume at full inspection (L), volume at full expiration (L), and volume error (%) (Table 5). The CT and FEM models before the simulation had identical volumes, with a mean (standard deviation) of 4.83 (0.68). The volumes of the FEM and CT models after the simulation (standard deviation) were 2.33 (0.56) and 0.48, respectively. Finally, the volume error of the CT and FEM overlap models was between 1.29 and 9.23%, with an average of 6.14% and standard deviation of 2.83.

### Quantitative and qualitative validation

#### Histogram of landmark relative error distributions

**Fig. 6:**
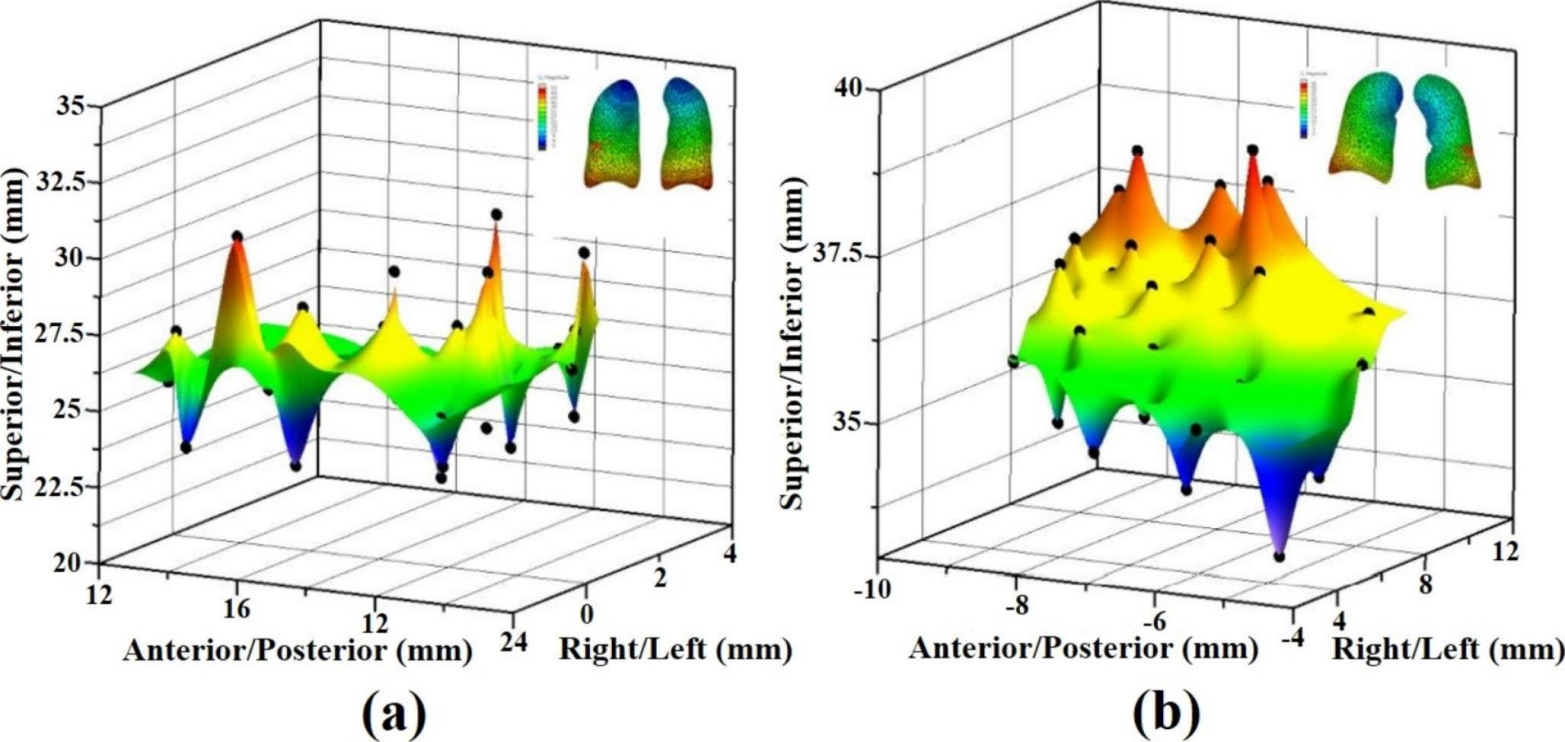
(**a**) Lung tumor landmark simulation results in Case 2 (L/R, A/P, S/I direction displacement results); (**b**) Lung tumor landmark simulation results in Case 4 (L/R, A/P, S/I direction displacement results)

To evaluate the effect of the rib, mediastinum, and diaphragmatic kinematics on lung bronchi (or tumor) movement, CT images of two selected individuals (Cases 2 and 4) were verified according to the modeling procedure in Fig. 1. Biomechanical model Cases 2 and 4 took landmarks in the EI state, simulated them in the EE state, and verified them with the CT images. Figure 6(a) and (b) show the simulation results for the individuals in Cases 2 and 4, respectively, taking 28 and 30 landmarks in the right lower (RL) and left lower (LL) lobes. After comparing the CT mean displacement (mm) of Cases 2 and 4 in Table 4, it was possible to calculate the error (%) values in the left/right (L/R), anterior/posterior (A/P), and superior/inferior (S/I) directions.

#### Specific pulmonary parenchymal trajectory

**Fig. 7:**
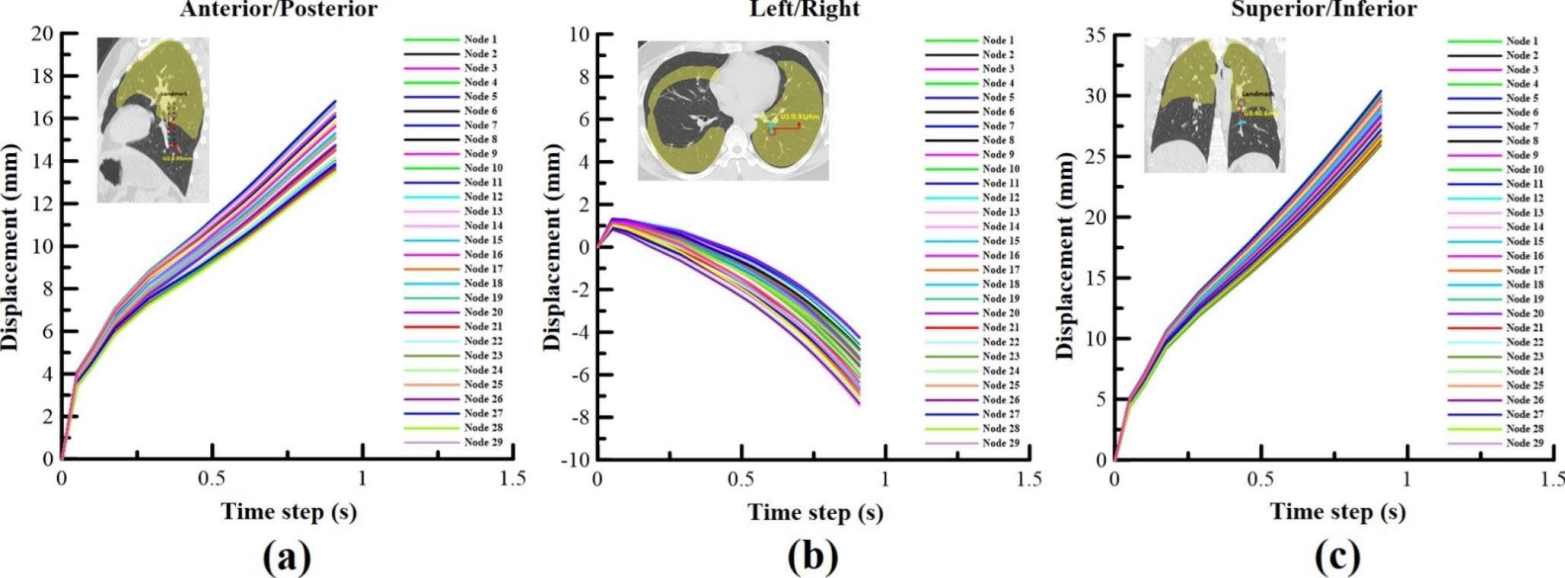
(**a**) Lung bronchi landmark simulation results in Case 4 (L/R direction displacement results; (**b**) Case 4 (A/P direction displacement results; (**c**) Case 4 (S/I direction displacement results

To confirm the location of the lung bronchi (or tumor) at each step of the simulation, the landmark coordinates on the branch of the bronchioles on the CT scan were moved to the node coordinates in the FEM program. Changes in lung bronchi (or tumor) motion were also assessed. Figure 7(a), (b), and (c) showed the displacement motion of a 29-node lung bronchi (or tumor) in the left/right (L/R), anterior/posterior (A/P), and superior/inferior (S/I) directions. As shown in Table 4, the average displacements of the simulation results were L/R: -5.91, A/P = 15.55, and S/I: 27.6 mm. Compared with the CT mean displacement data, the errors were 0.3%, 2.9%, and 1.0%, respectively. When the lungs contracted, due to transpulmonary pressure (EE), the displacement in the A/P direction was the most inaccurate, followed by the S/I and L/R directions.

**Table 5:** Volumetric analysis result

Subject no.	Gender	Volume at full inspiration (L)	Volume at full expiration (L)		Volume error (%)
		CT/FEM	CT	FEM	CT/FEM
Case1	M	5.33	3.10	3.14	1.29
Case2	F	3.57	1.64	1.78	8.53
Case3	M	5.44	2.72	2.87	5.51
Case4	M	5.51	1.95	2.13	9.23
Case5	M	4.92	2.48	2.30	7.25
Case6	M	4.92	3.29	3.10	5.77
Case7	F	3.99	2.22	2.25	1.35
Case8	M	5.53	1.95	2.12	8.71
Case9	M	4.25	1.70	1.83	7.64
Mean (SD)		4.83(0.68)	2.33(0.56)	2.39(0.48)	6.14(2.83)

#### Volumetric and relative error (cross-sectional area)

**Fig. 8:**
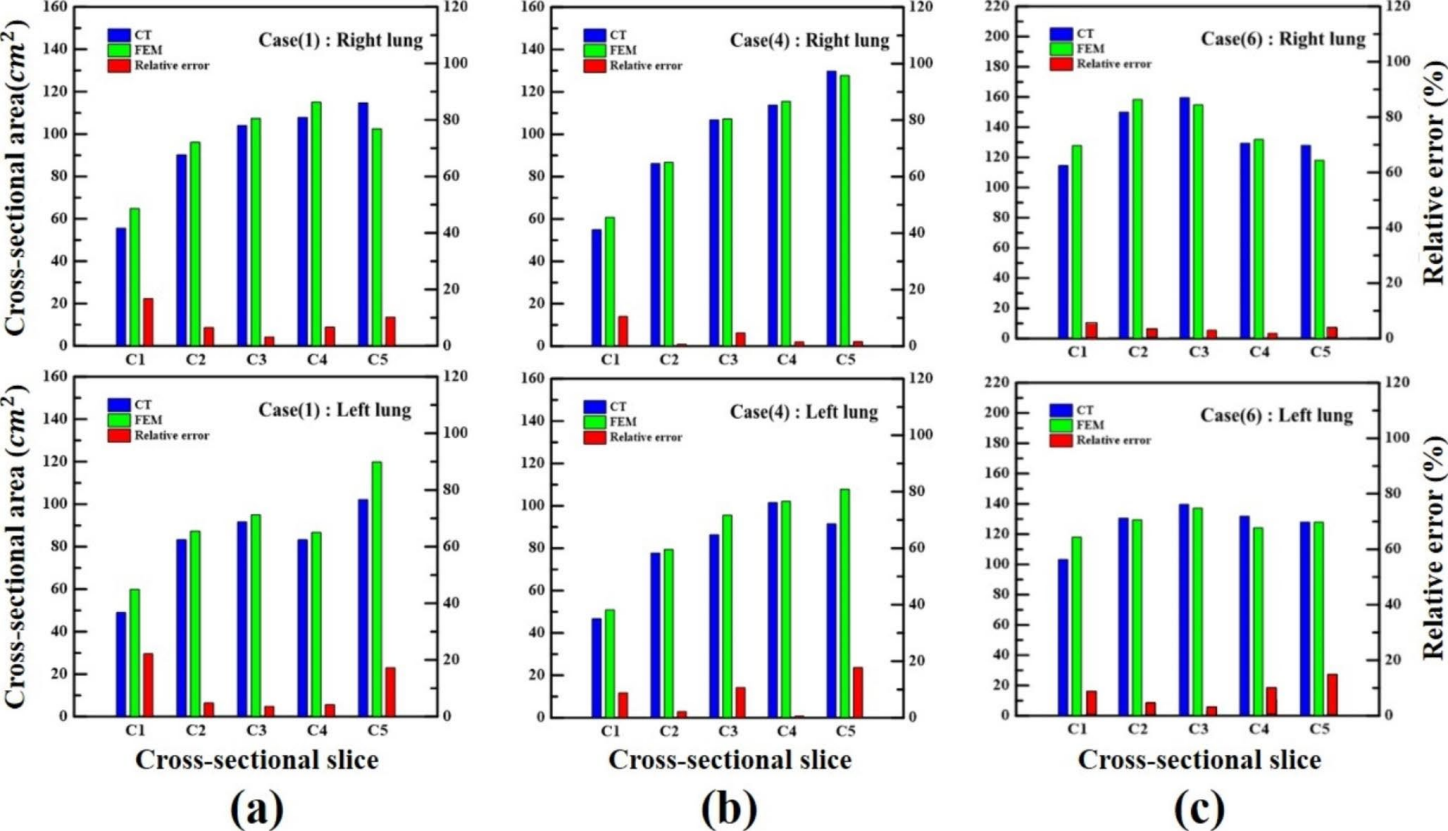
(**a**) Superior-inferior cross-sectional relative area error in Case 1 (CT and FEM Results); (**b**) Case 4 (CT and FEM Results); (**c**) Case 6 (CT and FEM Results)

CT and FEM 3D overlap models showed that the volumes at full inspiration (L) and expiration (L) were measured in Table 5. To quantitatively verify the superior-inferior cross-section, cross-sections were made and quantified as (C1–5) in Fig. 5(b). The correlation between the volume data in Table 5 and the area data in Fig. 5(c) was identified. As a result of the calculation in Fig. 8(a) and Table 5 (Case 1), the volume at full expiration (L) of the CT model was 3.1 L and the simulated FEM model was 3.14 L, which had the most accurate error of 1.29%.

The area errors of the upper lobes (C1–2) of the left lung were 22.18% and 6.51%, respectively. The middle lobe (C3) was 3.61%, and the lower lobes (C4–5) were 4.23% and 17.25%, respectively. The upper lobe of the right lung (C1–2) showed 16.75% and 6.52% errors, the middle lobe (C3) showed 3.17% errors, and the lower lobes (C4–5) showed 6.67% and 10.20% errors, respectively. The total mean relative area error was 8.66% and 10.75% for the right and left lungs, respectively.

As a result of the calculation in Fig. 8(b) and Table 5 (Case 4), the volume at full expiration (L) of the CT model was 1.95 L and the simulated FEM model was 2.13 L, which was the most inaccurate error of 9.23%. The area errors of the upper lobes (C1–2) of the left lung were 8.78% and 2.17%, respectively. The middle lobe (C3) was 10.63%, and the lower lobes (C4–5) were 0.54% and 17.73%, respectively. The upper lobe of the right lung (C1–2) showed 10.45% and 0.66% errors, the middle lobe (C3) showed 4.68% errors, and the lower lobes (C4–5) showed 1.49% and 1.61% errors, respectively. The total mean relative error was 3.77% and 7.97% for the right and left lungs, respectively.

As a result of the calculation in Fig. 8(c) and Table 5 (Case 6), the volume at full expiration (L) of the CT model was 3.29 L and the simulated FEM model was 3.1 L, which had a median error of 5.77%.

The area errors of the upper lobes (C1–C2) of the left lung were 9.79% and 6.65%, respectively. The middle lobe (C3) was 5.70%, and the lower lobes (C4–5) were 10.43% and 15.72%, respectively. The upper lobe of the right lung (C1–2) showed errors of 7.39% and 1.68%, the middle lobe (C3) was 1.43%, and the lower lobes (C4–5) were 0.98% and 4.45%, respectively. The total mean relative error was 3.18% and 9.65% for the right and left lungs, respectively.

Therefore, the relative error of the left lung was larger than that of the right lung. This indicates that deformation occurs as the lungs contract with boundaries and support, owing to the influence of the heart and liver. The relative error showed a significant difference in the upper and lower lobes of the lung, with the largest error observed in the upper lobe. Additionally, the trachea deforms as the lungs expand, and accordion-like elongation and contraction phenomena were also identified in the CT and FEM results. Finally, regarding the correlation between the cross-sectional data of the lung and volume data, it was found that the larger the error of the volume, the larger the relative error of the area. It was found that a proportional relationship exists.

## Discussion

In this study, it was difficult for clinicians to determine the location of lung lesions that perform complex breathing movements during minimally invasive surgery [36]. Therefore, to predict in advance, a specific model from CT was taken, the physical properties of the human lung were applied, and the FEM based on the analysis results was compared and verified with CT data to define the landmark relative error distribution, specific person’s lesion trajectory, and volumetric relative errors.

The results of these simulation data can be used to extract 3D models of the pulmonary anatomical structure that are suitable for decision-making and preoperative simulation in various lung disease surgical procedures [37]. It is also possible to predict the movement of the area of interest. Recently, dynamic 3D-CT technology (Synapse Vincent, Fuji Film Co., Ltd., Tokyo, Japan) has been developed and introduced in the field of thoracic surgery [38]. This technology provides high-speed, high-quality 3D images that help surgeons to more accurately understand the lung structure and plan the surgery. In addition, 3D navigation tool can be used to accurately segment structures, preserve interlobar veins, remove target tissue, and secure surgical margins during the surgery [39].

The following are clinical cases that have successfully used this technology to guide surgery or treatment of lung diseases:

- Lung cancer resection: The journal of Thoracic Disease in 2017, Duilio Divisi et al. used 3D MIS to guide the resection of lung tumors in 10 patients. The researchers found that the 3D MIS technique was able to accurately identify the location and extent of the tumors, and it allowed the surgeons to perform the resections with greater precision and less risk of complications [40].
- Lung nodule ablation: The journal of Thoracic Disease in 2022, Dazhi Pang et al. used 3D MIS to guide the ablation of lung nodules in 120 patients. The researchers found that the 3D MIS technique was able to accurately identify the location and size of the nodules, and it allowed the surgeons to perform the ablations with greater precision and less risk of complications [41].
- Emphysema treatment: The Chest journal in 2021, Karin Klooster used 3D MIS to guide the implantation of endobronchial valves in 5 patients with emphysema. The researchers found that the 3D MIS technique was able to accurately identify the location of the target airways, and it allowed the surgeons to implant the valves with greater precision and less risk of complications [42].
- Pulmonary fibrosis treatment: The Clinical Respiratory Journal in 2023, Megyesfalvi Zsolt used 3D MIS to guide the treatment of pulmonary fibrosis with photodynamic therapy. The researchers found that the 3D MIS technique allowed them to deliver the therapy more precisely to the affected areas of the lung, and it resulted in better clinical outcomes for the patients [29].
- Lung transplant: The journal General Thoracic and Cardiovascular Surgery in 2017, Chen-Yoshikawa Toyofumi F used 3D MIS to guide the transplantation of lungs from living donors. The researchers found that the 3D MIS technique allowed them to perform the transplantations with greater precision and less risk of complications [43].

The development of minimally invasive surgery (MIS) using these 3D models is expected to help improve the success rate of lung disease surgery and shorten the recovery period for patients after surgery.

## Conclusion

For a successful and accurate minimally invasive surgery, we performed a comparative analysis of lung deformation simulations during human respiration based on CT. Based on the biomaterial analysis, the Ogden_N = 6 model, among the 15 lung mechanical property models, was the most consistent with the data of the biaxial experiment, and the simulation results were also very accurate [44]. Compared with the CT and FEM models, the most deformed lobe among the upper, middle, and lower lobes during pulmonary respiration was the lower lobe, involving the diaphragm of the left lung. The lobe with the least deformation was the upper lobe of the right lung, and the error between the upper and middle lobes of the lungs was insignificant. In particular, in the S/I direction, which is the largest deformation, the lungs of the nine experimenters had large deformations from 23.60 to 78.05 mm, followed by A/P and L/R, and the relative error was the lowest in the S/I direction, followed by L/R and A/P.

The nine experimenters had a higher left lung error than a right lung error. We confirmed that the landmark relative error of the L/R, S/I, A/P direction of the lung lesion was well matched within the range of 1.1–2.67%. Finally, the volumetric relative error of the CT and FEM overlap models was between 1.29 and 9.23%, with an average of 6.14% and a standard deviation of 2.83%.

Human lungs make complex movements while in contact with many organs, bones, diaphragms, and blood vessels when breathing. In order to simulate these movements, the loads and boundary values ​​applied to the lungs were analyzed separately as transpulmonary pressure and boundary conditions. In order to overcome this, the mediastinum, ribs, trachea, and diaphragm, which have a large impact on displacement and deformation due to boundaries and supports, were added to the simulation for higher accuracy than previous studies. In addition, 9 cases were taken using the data that fit best among 15 hyperelastic material properties based on the curve fitting results of the existing experimental literature. However, it is difficult to perfectly understand the nonlinear biomaterial and mechanical properties of the lungs. Additionally, some errors occur during the lung segmentation and modeling process. In addition, it takes a lot of computational cost and time to predict lesions using the finite element model. Nevertheless, it has value in that it can be used as a tool for doctors to find the location of lesions during surgery. It also has a high potential for development into interdisciplinary research between thoracic surgery (human big data through machine learning) [45]. Therefore, in future studies, we will conduct research combining the finite element method and artificial intelligence. We will code the image segmentation, boundary and load conditions, etc., and verify them with clinical data. By doing so, doctors in the medical field will be able to treat patients more quickly than with the finite element analysis method by getting the computational results from the processor in a short time.

## References

[1] Song Y, Park C (2018). Pulmonary subsolid nodules: an overview and management guidelines. J Korean Soc Radiol.

[2] Krimsky W (2014). Thoracoscopic detection of Occult Indeterminate Pulmonary Nodules using Bronchoscopic Pleural Dye Marking. J. Community Hosp. Intern Med Perspect.

[3] Chen Y (2007). CT-guided Hook Wire localization of Subpleural Lung Lesions for Video-assisted thoracoscopic surgery (VATS). J Formos Med Assoc.

[4] Kuo S (2019). Electromagnetic Navigation Bronchoscopy localization versus percutaneous CT-guided localization for lung resection via video-assisted thoracoscopic surgery: a propensity-matched study. J Clin Med.

[5] Lee J (2019). Planting Seeds into the lung: image-guided percutaneous localization to Guide minimally invasive thoracic surgery. Korean J Radiol.

[6] Dawson L, Jaffray D (2007). Advances in image-guided Radiation Therapy. J Clin Oncol.

[7] Ehrhardt J (2007). An Optical Flow based Method for Improved Reconstruction of 4-D CT Data sets acquired during free breathing. Med Phys.

[8] Vedam S (2003). Acquiring a four-dimensional computed tomography dataset using an External Respiratory Signal. Phys Med Biol.

[9] McClelland J (2006). A continuous 4-D motion model from multiple respiratory cycles for Use in Lung Radiotherapy. Med Phys.

[10] Berger L (2016). A poroelastic model coupled to a Fluid Network with Applications in Lung modeling. Int J Numer Meth Biomed Eng.

[11] Shirzadi Z, Naini A, Samani A (2012) Lung tumor motion prediction during lung brachytherapy using finite element Model. Proc Med Imaging 2012: Image Guided Procedures Robotic Interventions Model 8316(1). 10.1117/12.906511

[12] Tehrani J (2015). Sensitivity of Tumor Motion Simulation Accuracy to Lung Biomechanical modeling approaches and parameters. Phys Med Biol.

[13] DeCarlo D (1955). Integrating anatomy and physiology for Behavior modeling. Med Meets Virtual Real.

[14] Werner R, Ehrhardt J, Schmidt R, Handels H (2009). Patient-specific finite element modeling of respiratory lung motion using 4D CT Image Data. Med Phys.

[15] Zhang T, Orton N, Mackie R, Paliwal B (2004). Technical note: a Novel Boundary Condition using contact elements for finite element deformable image Registration. Med Phys.

[16] Villard P, Beuve M, Shariat B, Baudet V, Jaillet F (2005). Simulation of Lung Behaviour with Finite Elements: influence of bio-mechanical parameters. Third Int Conf Med Inform Visualization - BioMedical Visualization Lond.

[17] Karami E, Gaede S, Samani TYL (2015). A biomechanical approach for in vivo lung tumor motion prediction during external beam radiation therapy. Image-Guided Procedures Robotic Interventions and Modeling.

[18] Fuerst, B et al (2015) Patient-specific Biomechanical Model for the prediction of lung motion from 4-D CT images. IEEE Trans Med Imaging. 34(2):599–607. 10.1109/TMI.2014.2363611 10.1109/TMI.2014.2363611 25343757

[19] Kubilay Muhammed Sünne, Ahmet Alka (2022). Lung cancer detection by using probabilistic majority voting and optimization techniques. Int J Imaging Syst Technol.

[20] Al-Mayah A, Moseley J, Brock K (2008). Contact surface and material nonlinearity modeling of human lungs. Phys Med Biol.

[21] Hamid L, Michael B, Philippe G, Behzad A (2021). Towards non-invasive lung tumor tracking based on Patient Specific Model of Respiratory System. IEEE Trans Biomed Eng.

[22] Abbas S (2011). Measurement of Lung Hyperelastic Properties using inverse finite element Approach. IEEE Trans Biomed Eng.

[23] Tuncer SA, Ahmet Alkan (2019).

[24] Doyle B et al (2015) Computational biomechanics for Medicine: New Approaches and New Applications. Springer. 10.1007/978-3-319-15503-6

[25] Behr M, Prs J, Llari M, Godio Y, Jammes Y, Brunet C (2010). A threedimensional human trunk model for the analysis of respiratory mechanics. J Biomech Eng.

[26] Palo M (2011). Finite element studies of the mechanical behaviour of the diaphragm in normal and pathological cases. CMBBE.

[27] Kimpara H (2005). Development of a three-dimensional finite element chest model for the 5th Percentile Female. Stapp Car Crash.

[28] Giroux M (2017). Patient-specific biomechanical modeling of the lung tumor for radiation therapy. Comput Methods Biomech Biomed Eng.

[29] Megyesfalvi, Zsolt (2023). Clinical insights into small cell lung cancer: Tumor heterogeneity, diagnosis, therapy, and future directions. ACS Journals.

[30] Al-Mayah A, Moseley J, Velec M, Brock K (2009). Sliding characteristic and material compressibility of human lung: Parametric study and verification. Med Phys.

[31] Zahra S, Ali SN, Abbas S (2013). Toward in vivo lung’s tissue incompressibility characterization for tumor motion modeling in radiation therapy. Med Phys.

[32] Adil A, Joanne M, Mike V, Kristy B (2011). Toward efficient biomechanical-based deformable image registration of lungs for image-guided radiotherapy. Phys Med Biol.

[33] Ladjal H et al (2015) Physiological and biomechanical model of patient specific lung motion based on 4D CT images. in Proc. 8th IEEE Biomed. Eng. Int. Conf. Thailand. 10.1109/BMEiCON.2015.7399567

[34] Ladjal H et al (2015) Biomechanical modeling of the respiratory system: human diaphragm and thorax. Computational biomechanics for Medicine New Approaches and New Applications. Springer, pp 101–115. 10.1007/978-3-319-15503-6_10

[35] Ozhasoglu C, Murphy MJ (2002) Issues in respiratory motion compensation during external-beam radiotherapy. Int J Radiat Oncol Biol Phys 52(5):1389–1399. 10.1016/S0360-3016(01)02789-4 10.1016/s0360-3016(01)02789-4 11955754

[36] Fegan KL et al (2022) Design and Simulation of the Biomechanics of Multi-Layered Composite Poly (Vinyl Alcohol) coronary artery grafts. Front Cardiovasc Med 9. 10.3389/fcvm.2022.883179 10.3389/fcvm.2022.883179 PMC9272978 35833186

[37] Giroux M, Ladjal H, Beuve M, Giraud P, Shariat B (2017). Patient-specific Biomechanical modeling of the lung tumor for Radiation Therapy. Comput Methods Biomech BioMed Eng.

[38] Chen-Yoshikawa Toyofumi F (2020). Current trends in thoracic surgery. Nagoya J Med Sci.

[39] Wu WB et al (2016) Three-dimensional computed tomography bronchography and angiography in the preoperative evaluation of thoracoscopic segmentectomy and subsegmentectomy. Journal of thoracic disease (image-Guided management of Lung Diseases). 9(8):S710–S715. 10.21037/jtd.2016.09.43 10.21037/jtd.2016.09.43 PMC5179351 28066674

[40] Divisi D et al (2017) Three-dimensional video-assisted thoracic surgery for pulmonary resections: an update. J Thorac Disease 3(79). 10.21037/jovs.2017.04.07 10.21037/jovs.2017.04.07 PMC5638256 29078642

[41] Dazhi, Pang (2022). 3D localization based on anatomical landmarks in the treatment of pulmonary nodules. J Thorac Disease.

[42] Karin Klooster and Dirk-Jan Slebos (2021). Endobronchial valves for the treatment of Advanced Emphysema. Chest J.

[43] Chen-Yoshikawa Toyofumi F, Date Hiroshi (2017). Three-dimensional image in lung transplantation. Gen Thorac Cardiovasc Surg.

[44] Yamada H (1970). Strength of Biological materials.

[45] Sunnetci KM, Kaba E, Celiker FB, Alkan A (2023) Deep Network-Based Comprehensive Parotid Gland Tumor Detection, Academic Radiology. 10.1016/j.acra.2023.04.028. S1076-6332 10.1016/j.acra.2023.04.028 37271636

